# Flexural Behavior of Orthotropic Steel–LUHPC Composite Bridge Decks: Experimental and Numerical Study

**DOI:** 10.3390/ma18092106

**Published:** 2025-05-03

**Authors:** Zebene Worku, Muyu Liu, Xin Wang, Guangzu Sheng

**Affiliations:** 1 School of Civil Engineering and Architecture, Wuhan University of Technology, Wuhan 430070, China; zebexwork@gmail.com (Z.W.);; 2 Hubei Key Laboratory of Roadway Bridge & Structure Engineering, Wuhan University of Technology, Wuhan 430070, China; 3 Wuhan Urban Construction Group, Wuhan 430068, China

**Keywords:** orthotropic steel–lightweight ultra-high-performance concrete composite bridge deck (OS-LUHPC-CBD), lightweight ultra-high-performance concrete (LUHPC), flexural behavior, experimental study, finite element analysis

## Abstract

Orthotropic Steel Bridge Decks (OSBDs) are often used in long-span bridges due to their high performance and ease of installation. However, issues such as fatigue cracking and the deterioration of asphalt overlays due to their local stiffness inefficiency necessitate innovative solutions. Orthotropic Steel–Ultra-High-Performance Concrete Composite Bridge Decks (OS-UHPC-CBDs) have enhanced OSBD performance; however, they have disadvantages such as a heavier weight and high initial cost requirements. In this study, an Orthotropic Steel–Lightweight Ultra-High-Performance Concrete Composite Bridge Deck (OS-LUHPC-CBD) is proposed as a solution that integrates a novel Lightweight Ultra-High-Performance Concrete (LUHPC) with a high-strength Q425 steel deck and trapezoidal ribs. A comprehensive experimental investigation, including full-scale four-point bending tests, was undertaken to evaluate the flexural behavior of the proposed OS-LUHPC-CBD compared to the OS-UHPC-CBD. The experimental results show that the proposed OS-LUHPC-CBD has equivalent flexural capacity and improved ductility compared to the OS-UHPC-CBD. This study found the proposed OS-LUHPC-CBD to be a promising solution for application in long-span bridges with an 8.4% lighter weight and a 6.8% lower cost, and with the same ease of construction as OS-UHPC-CBDs. A finite element model with a strong correlation was developed and validated through the experimental results. Based on this, a parametric study was undertaken on the effect of the key geometric design parameters on the flexural capacity of the OS-LUHPC-CBD.

## 1. Introduction

Orthotropic Steel Bridge Decks (OSBDs) are commonly used in long-span bridges because of their higher strength and ease of construction with reduced superstructure bulk, thus decreasing the pressure on cables and towers [1,2,3,4]. OSBDs are typically made of conventional steel deck plates, transverse crossbeams, and longitudinal ribs [3,5,6], triggering local stiffness insufficiency, which seriously affects the safety [7] and durability [8,9] of long-span bridges. Fatigue cracking can occur after 10 to 20 years of operation and premature asphalt overlay degradation after 5 to 8 years of service [10,11,12,13].

To address these problems, the Orthotropic Steel–Ultra-High-Performance Concrete Composite Bridge Deck (OS-UHPC-CBD) was introduced and first tested on the Dutch Garland Bridge and fully implemented on the Gärtnerplatz Bridge in Germany in 2007 [14]. By delaying premature deterioration, the UHPC slab joined to the OSD with shear connectors increases flexural stiffness, lowers fatigue failure, and prolongs pavement service life [15,16,17]. UHPC also effectively prevents the local buckling and lateral torsional buckling of steel structures [16,17]. However, OS-UHPC-CBDs pose the problems of extra weight and high initial costs [16,18], and the aim of existing Lightweight Concrete Decks is primarily to reduce the thickness of Ultra-High-Performance Concrete (UHPC) slabs alone and they rely heavily on the stiffness of the Orthotropic Steel Deck (OSD). This overlooks the structural contribution of UHPC, particularly its ability to prevent local and lateral torsional buckling [18,19,20,21,22,23].

Therefore, in this study, we propose a super-lightweight OS-LUHPC-CBD, which integrates novel Lightweight Ultra-High-Performance Concrete (LUHPC) and high-strength steel (Q425) materials as a lightweight and cost-effective solution for long-span bridges with Orthotropic Steel Bridge Decks (OSBDs). The LUHPC introduced in this research is a novel material developed by the authors’ research team to overcome the limitations of Ultra-High-Performance Concrete (UHPC), specifically for long-span bridge deck applications [24,25,26,27,28]. Through rigorous testing and optimization, the LUHPC achieves a remarkable balance between lightweight design and excellent uniaxial tensile and compressive strengths, as well as great flexural and shear capacities. Its composition enhances stress distribution, which contributes to good resistance to cracking and reduces the likelihood of premature failure [29,30,31,32]. Additionally, the LUHPC demonstrates strong bonding with steel reinforcement [31], making it convenient for long-span bridge applications.

From an environmental perspective, LUHPC presents several sustainable advantages. It utilizes lightweight aggregates such as clay or recycled materials, thereby reducing the reliance on natural aggregate resources. In some cases, the energy consumed in the production of these lightweight aggregates is lower than that for conventional alternatives. Due to LUHPC’s high strength and durability, structural elements can be designed with reduced thicknesses, leading to significant material savings and reduced carbon emissions per unit volume. Furthermore, LUHPC’s high impermeability and corrosion resistance contribute to longer service lives, lower maintenance demands, and extended replacement cycles, collectively resulting in reduced lifecycle carbon emissions.

The long-term performance of LUHPC is a critical factor in determining its suitability for bridge infrastructure, particularly for long-span bridges exposed to aggressive environments and sustained loading. Durability testing has shown that LUHPC features exceptionally low chloride ion diffusion coefficients (2.4 × 10^−13^ to 3.9 × 10^−13^ m^2^/s), a freeze–thaw mass loss rate of less than 1%, and a negligible carbonation depth. These results correspond to high durability grades (RCM-V, F500, and T-V), confirming LUHPC’s resistance to corrosion, freeze–thaw degradation, and carbonation, which are essential for marine or cold-climate bridge environments [33].

In terms of time-dependent deformation, the creep testing of LUHPC specimens with a steel fiber content ranging from 2.0% to 3.0% indicated low creep coefficients (0.6–0.9). These low values suggest that LUHPC retains dimensional stability over time, further supporting its application in continuous or prestressed bridge components [28]. Furthermore, with respect to shrinkage behavior, shrinkage-compensating techniques have proven highly effective. The use of calcium sulfoaluminate-based expansive agents, coupled with pre-wetted lightweight aggregates, significantly mitigates shrinkage by enhancing internal curing and reducing the “water competition” effect. The synergistic action of steel fibers, expansive agents, and internal curing agents leads to a substantial reduction in autogenous shrinkage, which is a known concern in dense UHPC matrices [27].

LUHPC also has good thermal resistance, mainly because of the aggregates used in its design mix. These aggregates often include materials such as clay that naturally resist heat. Additionally, LUHPC has a very dense and compact structure that slows down the transfer of heat. Its low porosity also helps prevent heat from moving through it easily. These features together make LUHPC effective in handling high temperatures and protecting structures from heat damage.

This study investigates the flexural behavior of the proposed OS-LUHPC-CBD in comparison with OS-UHPC-CBD through experimental investigation and numerical simulations. A comprehensive experimental investigation using full-scale four-point bending tests was undertaken to evaluate the proposed OS-LUHPC-CBD system’s flexural performance in comparison with the OS-UHPC-CBD. Four composite bridge deck specimens were fabricated and tested. The failure modes, load–displacement behavior, ductility, interfacial slip at the steel–concrete interface, and strain distribution are the important flexural behavior performance factors that have been thoroughly investigated. The system has mainly shown flexural failure and ductile flexural behavior under ultimate loads. A finite element model [8] that incorporates concrete damage plasticity (CDP) was developed to predict the flexural behavior of the OS-LUHPC-CBD and OS-UHPC-CBD systems. This model addresses the limitations of current finite element models, specifically in capturing the progressive damage of LUHPC and UHPC materials and the non-uniform stress distribution along ribbed steel decks [34,35,36]. By precisely capturing the progressive damage and stress redistribution within the steel–LUHPC system, the model was validated against experimental data and demonstrated high correlation and reliability. Utilizing the validated finite element model, a parametric study was conducted to explore the effects of key geometric design parameters on the flexural capacity of the OS-LUHPC-CBD and OS-UHPC-CBD systems. The findings from the experimental investigations and numerical analysis show that the proposed OS-LUHPC-CBD is a promising solution for application in long-span bridges.

## 2. Experimental Program

### 2.1. Specimens for the Flexural Tests

**Figure 1:**
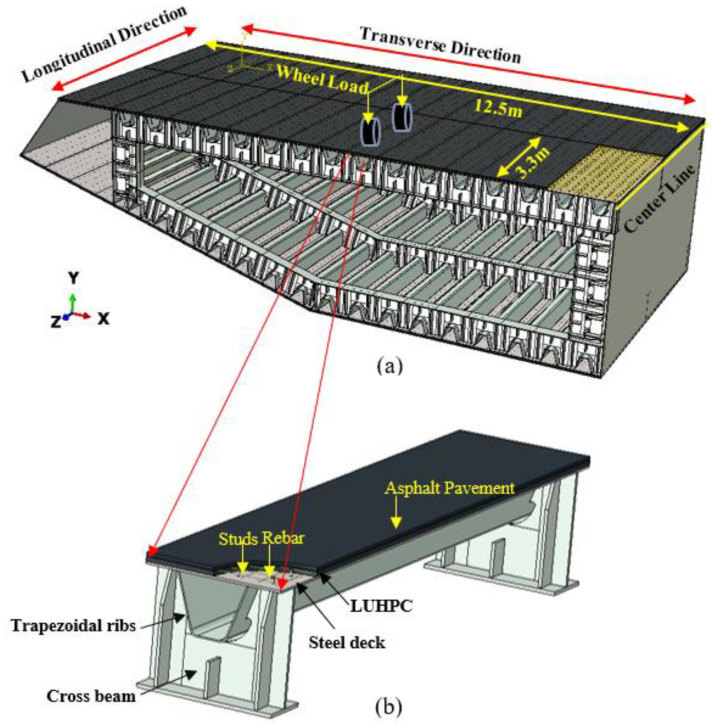
(**a**) Typical configuration of a long-span bridge steel box girder; (**b**) proposed OS-LUHPC-CBD as part of the long-span bridge box girder.

The proposed composite bridge decks are designed for applications in long-span bridge steel box girders, a typical configuration of which is shown in Figure 1. The composite bridge decks are supported by the crossbeams (floor beams) in the longitudinal direction of the bridge. The width of the composite bridge deck is 12.5 m and the spacing of the crossbeams is 3.3 m. Because the width is larger than the spacing, the wheel load is mainly transmitted to the crossbeams by the bending of the composite bridge decks in the longitudinal direction of the bridge. Therefore, the longitudinal flexural behavior of the proposed composite bridge decks requires investigation.

This experimental program evaluated and compared the flexural behavior of the proposed Orthotropic Steel–Lightweight Ultra-High-Performance Concrete Composite Bridge Deck (OS-LUHPC-CBD) and Orthotropic Steel–Ultra-High-Performance Concrete Composite Bridge Deck (OS-UHPC-CBD). The specimens used for testing were designed based on Level 1 principles and Level 2 simplified two-dimensional (2D) design procedures for Orthotropic Steel Deck (OSD) design [6,37].

**Figure 2:**
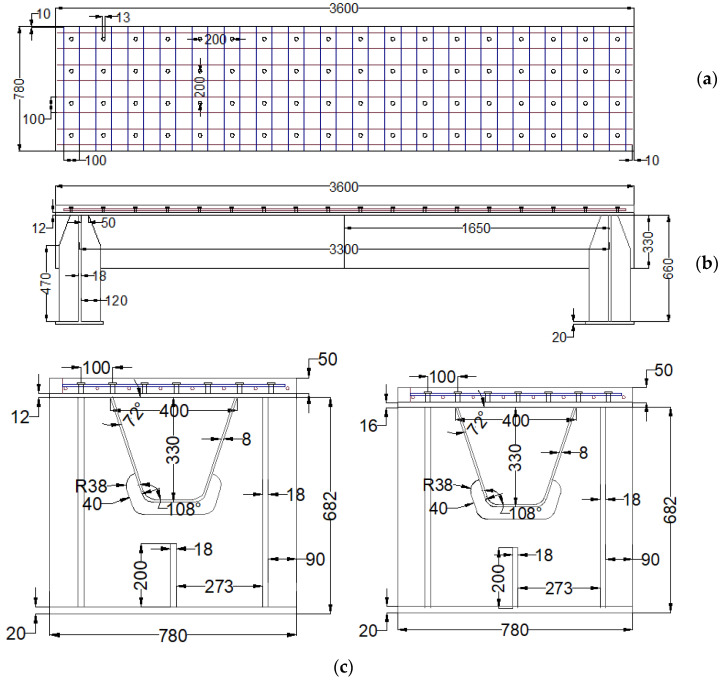
Detailed design of proposed specimens with closed trapezoidal rib. (**a**) Top view, (**b**) side view and (**c**) front view with 12 mm and 16 mm thick steel deck from left to right.

**Figure 3:**
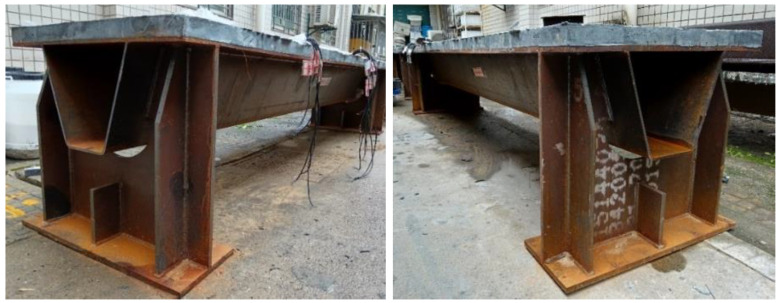
The proposed specimens constructed with 12 mm and 16 mm thick steel deck and 50 mm reinforced LUHPC slabs.

Four specimens were fabricated and tested to investigate their flexural behavior. The specimens had the same structure, but different orthotropic steel deck thicknesses and reinforced LUHPC and UHPC slabs, as illustrated in Figure 2. The proposed specimens after construction are shown in Figure 3. Each specimen was made of an orthotropic steel deck with a trapezoidal rib and LUHPC and UHPC slabs reinforced with longitudinal and transverse reinforcement bars, and were connected to an orthotropic steel deck with short shear studs. The effective span length of the specimens was 3300 mm, supported by floor beams (crossbeams) at both ends, as shown in the configuration of the long-span bridge steel box girder in Figure 1. The crossbeams at both ends were subjected to a four-point bending test to simulate wheel load application conditions and to ensure that no local or transverse buckling occurred in the fatigue-prone areas of the specimens, particularly at the rib-to-deck (RD) and rib-to-floor beam (RF) connections, which could affect the flexural behavior. The two configurations are as follows:

Configuration 1: Two OS-LUHPC-CBD specimens with closed trapezoidal ribs with 12 mm and 16 mm thick orthotropic steel decks and a 50 mm reinforced LUHPC slab.

Configuration 2: Two OS-UHPC-CBD specimens with closed trapezoidal ribs with 12 mm and 16 mm thick steel decks and a 50 mm reinforced UHPC slab.

The selection of orthotropic steel deck plates of 12 mm and 16 mm in thickness was based on standard engineering practice, where thicknesses of 12 mm, 14 mm, and 16 mm are typically used in bridge construction. These values represent the standard design range’s practical lower and upper bounds. Due to the complexity and resource-intensive nature of full-scale experimental testing, it was not feasible to test all possible thicknesses. Therefore, 12 mm and 16 mm were chosen as representative cases to capture the influence of deck plate thickness on structural performance.

### 2.2. Material Properties of LUHPC and UHPC

The UHPC employed in this study was a normal UHPC weighing 2800 kg/m^3^, which is very heavy for use in long-span bridges and has the additional disadvantages of a high initial cost and an adverse environmental impact. For this reason, UHPCs are still being researched in order to reduce the amount of carbon [38] and cement involved [39], optimize mix proportions and improve material utilization [40,41], and design new lightweight mixes [42,43,44].

As one of the state-of-the-art outcomes from the cutting edge research on the UHPC lightweight mix design, the LUHPC [24,25] employed in the proposed OS-LUHPC-CBD system in this research has a significantly lower density of 2045 kg/m^3^, enhancing its suitability for long-span bridge deck applications. The mix designs of both LUHPC and UHPC materials are provided in Table 1 and Table 2.

**Figure 4:**
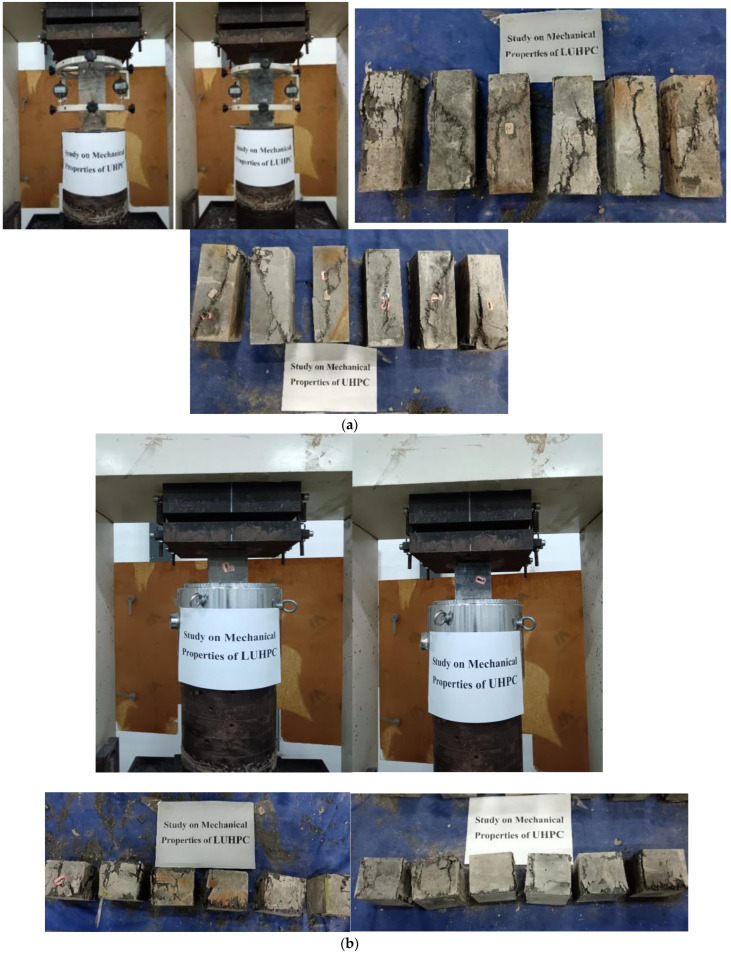

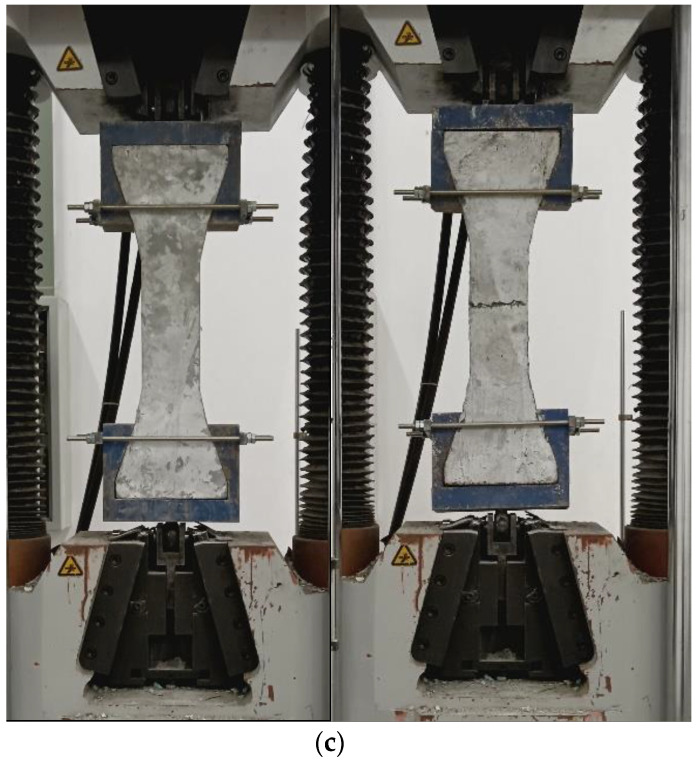
Mechanical properties of LUHPC and UHPC materials. (**a**) Elastic modulus, (**b**) compressive strength and (**c**) tensile strength.

The mechanical properties of the LUHPC and UHPC materials were investigated and determined based on the standard testing procedures outlined in the Chinese specification GB/T 31-387 [45]. Six prism specimens (Figure 4b) were tested for determining the elastic modulus; six cubic specimens (Figure 4a) were tested for determining the compressive strength; and six dog-bone-shaped specimens (Figure 4c) were tested for determining the tensile strength. The test setups and the specimens’ response after the test for both LUHPC and UHPC materials are shown in Figure 4a–c. The elastic modulus, compressive strength, and uniaxial tensile strength of both materials are presented in Table 3.

Q425 steel was used to fabricate the orthotropic steel deck (OSD) component within both the OS-LUHPC-CBD and OS-UHPC-CBD. The mechanical properties for the steel, reinforcement, and headed steel shear stud are shown in Table 4 based on the data from the fabricator.

### 2.3. Fabrication of Specimens

**Figure 5:**
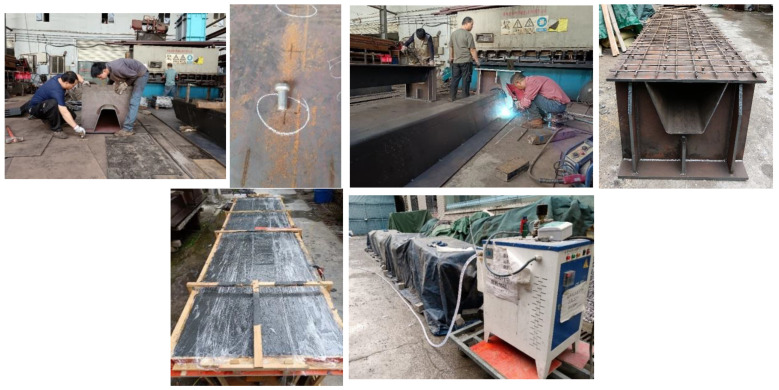
Fabrication processes of the specimens and steam curing of the slabs.

The fabrication process of the specimens is illustrated in Figure 5, including the steam curing phase. The fabrication followed five key steps. (1) Steel plate preparation: Steel plates were cut, assembled, welded, and prepared. (2) Shear connector welding: Studs were welded on top of the steel deck at predetermined intervals to serve as shear connectors. (3) Reinforcement placement: Longitudinal and transverse reinforcement bars were arranged according to the detailed design. (4) Concrete placement: The LUHPC and UHPC were cast to their respective specimens gradually and vibrated all over to ensure the best integration with the steel plates through the shear studs. (5) Curing procedure: To guarantee the best possible hydration of both UHPC and LUHPC slabs, the specimens were steam-cured for three days at 80 °C and 90% humidity after casting.

### 2.4. Test Setup, Loading Conditions, and Instrumentation

**Figure 6:**
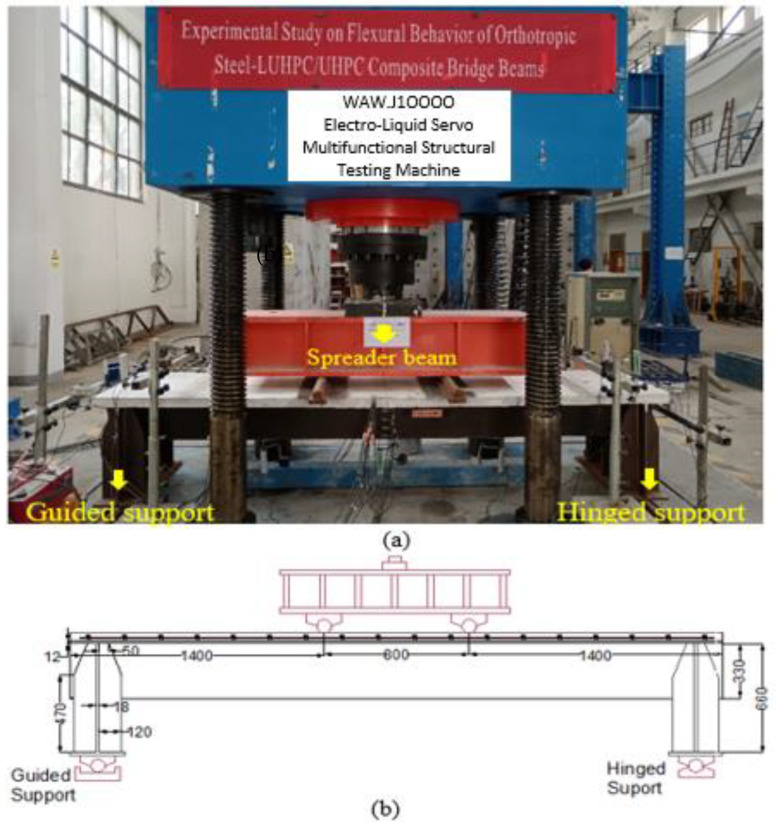
General four-point flexural test setup of the specimens: (**a**) after installation and (**b**) pictorial representation.

The specimens were tested under four-point bending to evaluate their flexural behavior. The test setup was similar for both specimens. Figure 6a,b depict the testing setup with rollers and a spreader beam. Two line loads were symmetrically applied to each specimen through a spreader beam generating a pure bending span (*L_b_*) in the middle.

**Figure 7:**
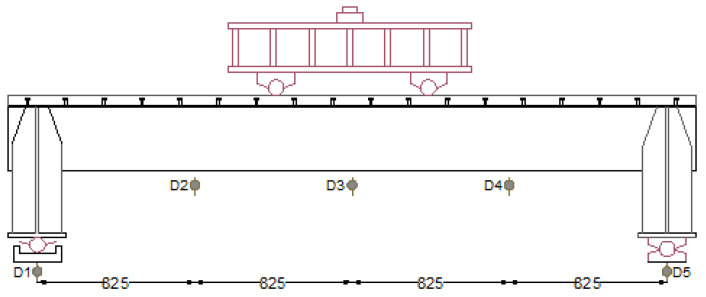
Dial indicators marked D_1_ to D_5_ used to measure the vertical deflections.

As can be seen in Figure 7, five dial indicators (D_1_ to D_5_) were installed in the specimens to assess vertical deflections. Equation (1) was employed to calculate the mid-span deflection (*D*) of the specimens.

(1) $$D=D_{3}-(D_{1}+D_{5})/2$$

**Figure 8:**
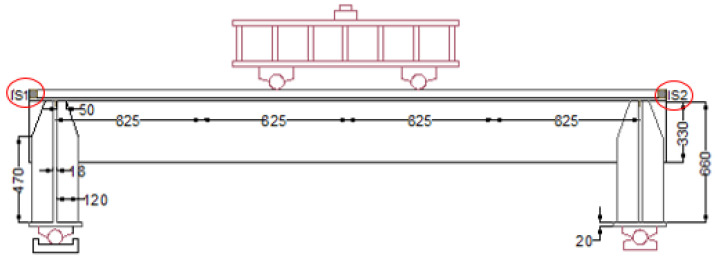
Dial indicators IS1 and IS2 for measuring slip between steel deck and slabs.

Dial indicators, designated IS1 and IS2 in Figure 8, were used to monitor the interfacial slip between the steel plate and the LUHPC and the UHPC slabs at both ends of the specimens and to measure the composite system’s bond performance under loading.

**Figure 9:**
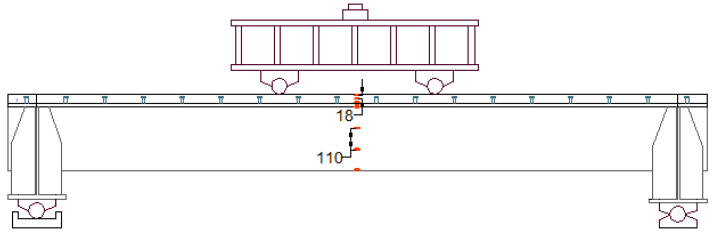
Strain gauges at the mid-span on the steel deck, ribs, and slab.

Electrical resistance strain gauges were positioned at points on the specimens to track strain distributions. These gauges were placed at the mid-span on the steel deck, the trapezoidal ribs, and the LUHPC and UHPC slabs. Understanding how the composite system behaves under loads requires knowledge of the strain distribution between the materials based on the strain gauges. Figure 9 shows the strain gauges’ location on the specimens’ mid-span cross section.

The load was applied by the actuator at the middle span, as shown in Figure 6a. Before the loading began, a preload of 30% of the total load was applied to the specimens. The preload was used to test the setup and instruments as well as to create and check contacts for the test setup. During the loading for the flexural test, the specimens were first loaded monotonically in the elastic stage while being controlled by force at loading rates of 20 kN/Min until the load–displacement curves began to show nonlinear behavior. Then, the specimens were loaded monotonically at a rate of 2 mm/min under displacement control following the elastic stage until the specimens failed. Finally, the test was terminated when the applied load began to decrease.

## 3. Experimental Results and Discussion

### 3.1. Failure Mode

The four specimens mainly exhibited flexural failure and ductile flexural behavior under the ultimate load. This involved the crushing of LUHPC and UHPC slabs and yielding of the trapezoidal rib at the pure bending span. These failure mechanisms align with earlier research on composite bridge decks that use steel–UHPC systems [17,46,47,48,49,50]. No crushing or visible surface damage was observed on the LUHPC or UHPC slabs until the inflection point was reached. However, cracks developed at both ends of the specimens on the crossbeam support region after the elastic stage, but did not influence the flexural behavior of the specimens as they appeared at the negative moment region.

With the increased applied load, the specimens underwent compressive crushing of the LUHPC and UHPC slabs and tensile yielding of the trapezoidal rib at the pure bending span, indicating the crushing failure of the specimens. The crushing failure occurred at 1251 kN and 1325 kN for the OS-LUHPC-CBD specimens and at 1254 kN and 1332 kN for the OS-UHPC-CBD specimens with the 12 mm and 16 mm steel decks, respectively. The OS-UHPC-CBD specimens showed negligibly greater peak loads than the OS-LUHPC-CBD specimens, which did not considerably affect system performance in the application. With its lighter weight and low cost, the LUHPC slab offers additional advantages [24], making the OS-LUHPC-CBD a competitive choice, particularly for the design of long-span bridges, since reducing weight can lessen structural demands and enhance overall efficiency.

There was no inter-layer separation or debonding between the concrete slabs and the steel deck plate in any of the specimens, which confirms that the shear connectors effectively maintain composite action. Additionally, no local buckling of the orthotropic steel plate was observed, which indicates that the steel deck’s structural integrity was preserved until failure in all specimens. Peeling of the slabs at the end of the specimens was also not observed in any of the OS-LUHPC-CBD or OS-UHPC-CBD specimens with 12 mm steel decks, but did appear in the OS-UHPC-CBD specimen with a 16 mm steel deck.

Interfacial slippage was observed at both ends of every specimen, emphasizing the importance of interface behavior in specimens. This phenomenon has been extensively studied, illustrating the vital role of shear connectors in preventing slippage and improving flexural performance [17,51]. Slippage can be minimized by utilizing alternative shear connection systems or optimizing the interface design between the steel deck and the concrete.

**Figure 10:**
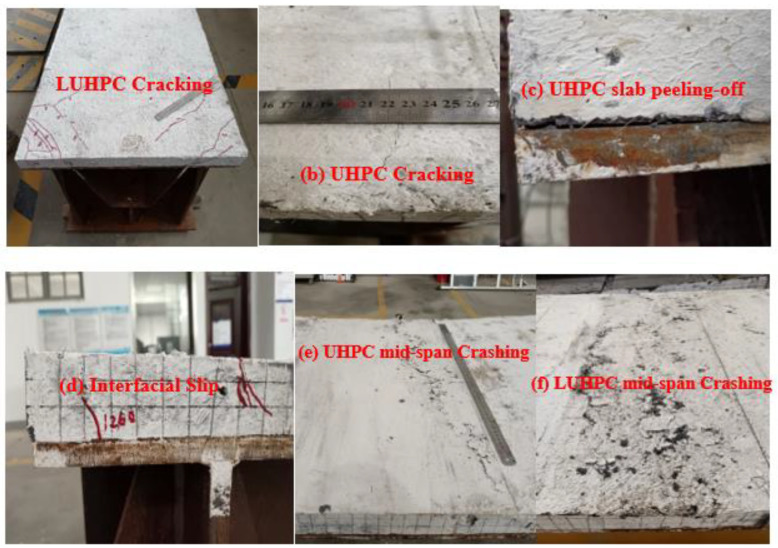
Failure process of the OS-LUHPC-CBD and OS-UHPC-CBD. (**a**) LUHPC cracking, (**b**) UHPC cracking, (**c**) UHPC slab peeling off, (**d**) interfacial slip, (**e**) UHPC mid span crashing, (**f**) LUHPC mid span crashing.

Generally, as can be depicted in Figure 10, the failure modes found in the experimental study were primarily characterized by flexural failure, evidenced by mid-span crushing of both LUHPC and UHPC slabs. The study demonstrated that all of the specimen configurations exhibited similar failure characteristics.

### 3.2. Load–Deflection Response

**Figure 11:**
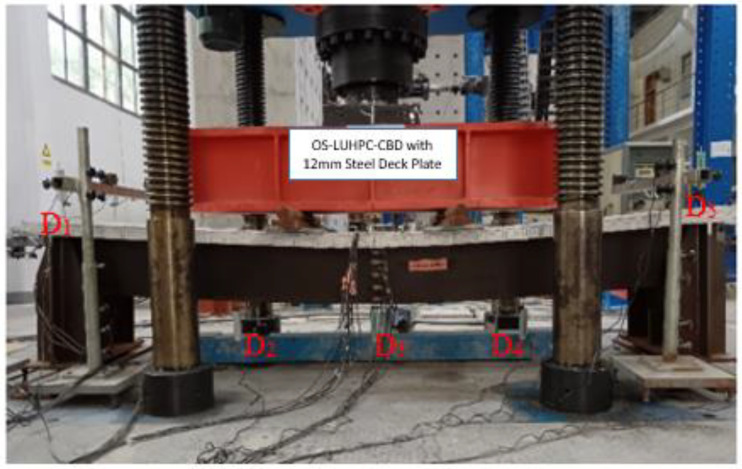
Dial indicators marked D_1_ to D_5_ installed to measure vertical deflections.

**Figure 12:**
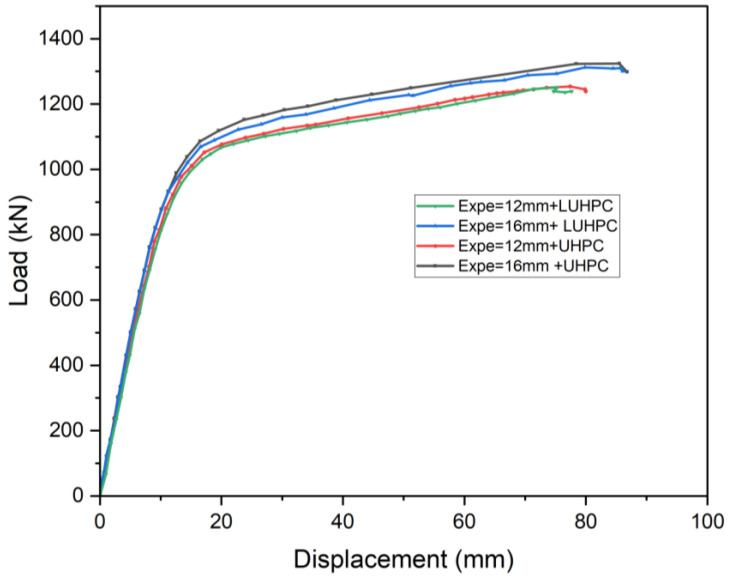
Load–deflection response of OS-LUHPC-CBD and OS-UHPC-CBD.

The load–deflection behavior of the OS-LUHPC-CBD and OS-UHPC-CBD specimens under increasing load was thoroughly observed, with the results illustrated in Figure 11 and Figure 12. The load–deflection behavior is divided into four distinct stages: elastic, elastoplastic, plastic, and failure. The elastic stage, where the response is linear, was observed at the initial loading. As the load increased, the specimens transitioned into an elastoplastic state due to plastic deformation in the steel of the trapezoidal ribs. An increase in deflection characterized this stage, while the system remained able to resist increasing loads. The plastic stage followed as concrete crushing was initiated at the mid-span, marking the commencement of failure. The specimen exhibited significant deflection at this point, but the applied load continued to increase until the concrete slab’s crush and failure occurred.

The OS-LUHPC-CBD specimens with 16 mm steel decks exhibited a yield load of 934 kN and an ultimate load of 1325 kN, while the OS-UHPC-CBD specimens with 16 mm steel decks demonstrated a yield load of 941 kN and an ultimate load of 1332 kN. The OS-LUHPC-CBD specimens with the 12 mm steel deck reached a yield load of 860 kN and an ultimate load of 1251 kN, while the OS-UHPC-CBD with the 12 mm steel deck reached a yield load of 872 kN and an ultimate load of 1254 kN, with minor differences indicating that the OS-LUHPC-CBD and OS-UHPC-CBD specimens have equivalent load-bearing capacity and flexural capacity.

At failure, both systems exhibited a typical crushing failure mode at mid-span, characterized by mid-span crushing of the LUHPC and UHPC slabs and yielding of the trapezoidal rib. The findings from this experimental investigation also show that when the steel deck thickness decreased from 16 mm to 12 mm in both the OS-LUHPC-CBD and OS-UHPC-CBD specimens, it resulted in a 6.2% and 5.9% decrease in the ultimate load-bearing capacity and 8.6% and 7.9% reduction in yield load, respectively, with no physically observable differences in their response upon the ultimate load application.

The findings from the load–deflection response also support the potential of the LUHPC slab to be the best composite component for forming OS-LUHPC-CBDs, particularly when weight reduction and cost savings without sacrificing structural performance are critical design considerations.

The proposed OS-LUHPC-CBD specimens with LUHPC slabs offer considerable advantages in terms of weight reduction and low cost, which is particularly advantageous in the design of long-span bridges. The OS-LUHPC-CBD is calculated to be 8.4% lighter in weight and 6.8% lower in cost than the OS-UHPC-CBD.

### 3.3. Ductile Performance

**Figure 13:**
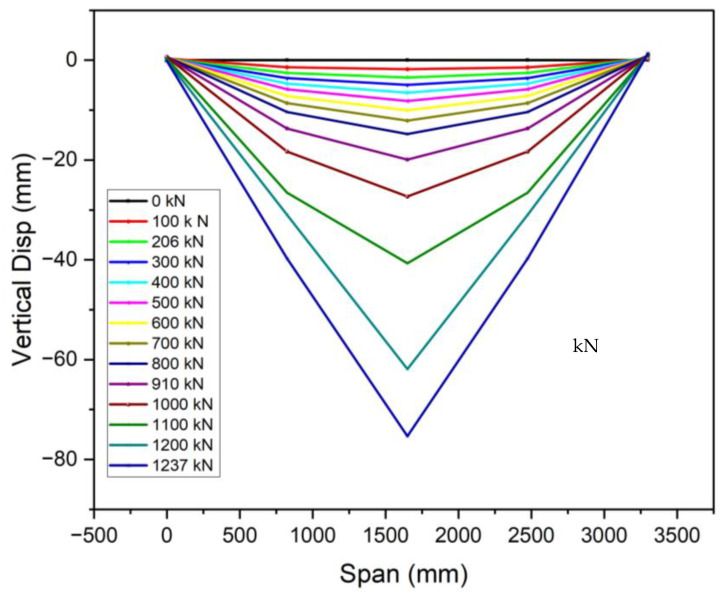
Vertical deflection development of the OS-LUHPC-CBD (12 mm steel deck).

**Figure 14:**
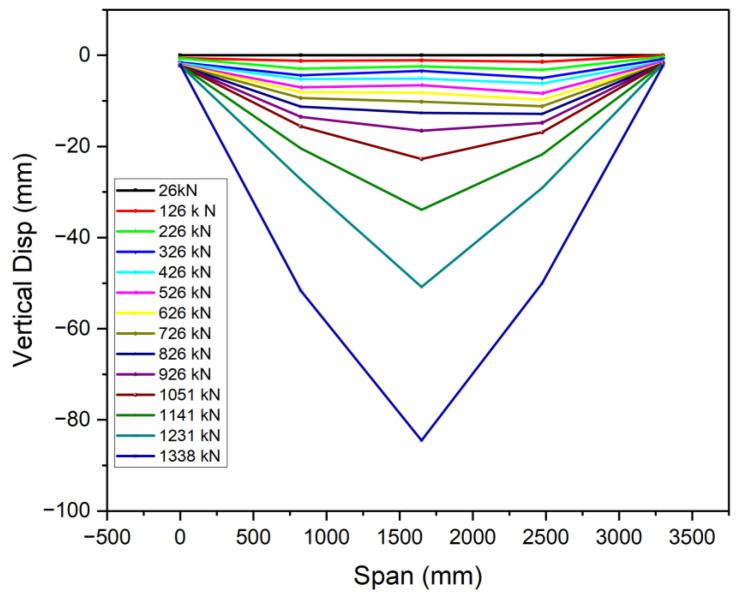
Vertical deflection development of the OS-LUHPC-CBD (16 mm steel deck).

**Figure 15:**
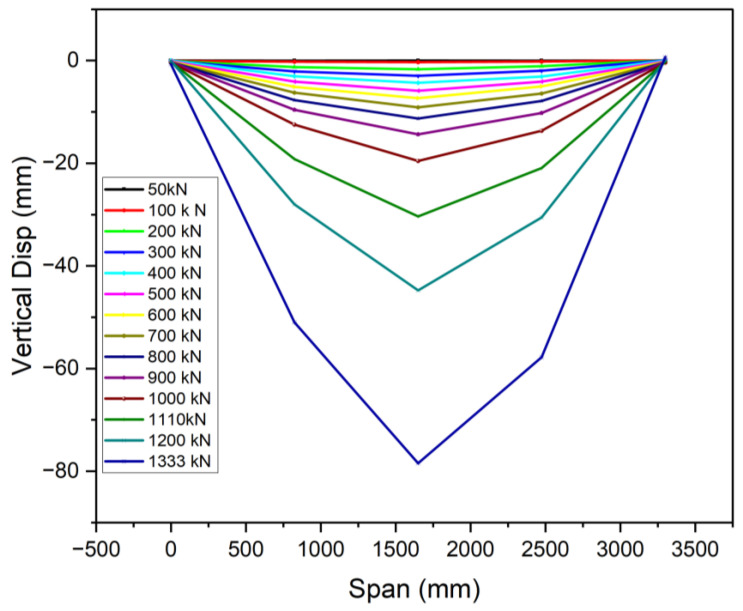
Vertical deflection development of the OS-UHPC-CBD (16 mm steel deck).

**Figure 16:**
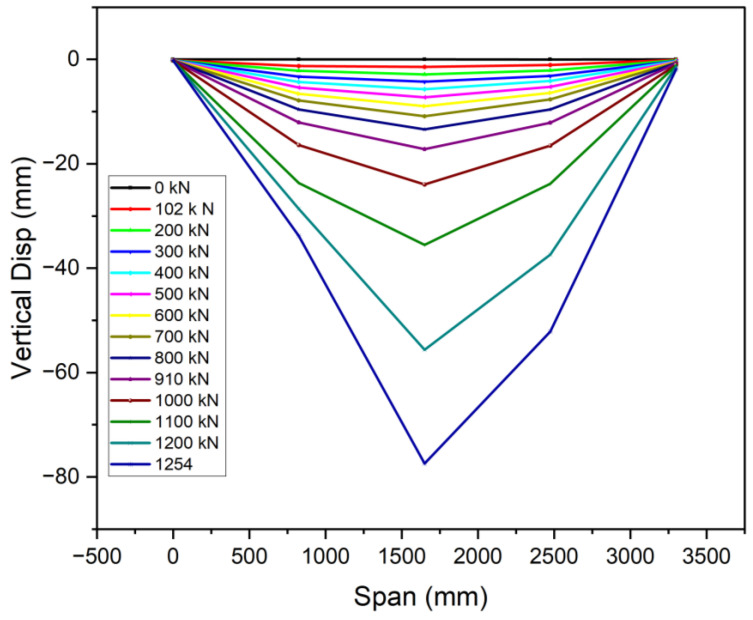
Vertical deflection development of the OS-UHPC-CBD (12 mm steel deck).

The recorded displacement gauges’ test results showed that the vertical deflections of all specimens were symmetric around the mid-span, in line with the principles of the four-point bending test. The specimens displayed symmetrical vertical deformation, as can be observed in Figure 13, Figure 14 and Figure 15. A minor irregularity was noted in the OS-UHPC-CBD with a 12 mm steel deck among the four specimens, as can be seen from Figure 16. In all of the specimens, a gradual vertical deflection was noted up to approximately 70% of their ultimate load-bearing capacity, after which the vertical deflection increased significantly, indicating the beginning of inelastic behavior.

The deflection ductility coefficient (μΔ) is an important measure of a structure’s capacity to sustain considerable deformation and energy dissipation without abrupt failure. It is characterized as the quotient of the maximum deflection at the ultimate load (Δu) to the maximum deflection at the yield load (Δy), and is commonly used to evaluate the deformation capacity and energy dissipation of composite structures [17]. As composite systems do not exhibit a clear yield point, it was determined as the point where the concrete’s compressive strain reached 1000 με, based on Li et al. (2021) [52].

The deflection ductility coefficients for the four specimens are presented in Table 5. The proposed lightweight OS-LUHPC-CBD specimens with 12 mm steel deck plates exhibited the highest ductility, and the OS-UHPC-CBD with a 16 mm steel deck plate had the lowest ductility coefficient. These values indicate that LUHPC performs better in terms of deformation capacity than UHPC, which can be attributed to its lower density and enhanced flexibility, which offers increased deformation tolerance in structural applications.

Both OS-LUHPC-CBD and OS-UHPC-CBD systems have energy dissipation capacity, which is crucial for understanding their long-term performance in real-world applications, where repeated cyclic loading can lead to fatigue and gradual degradation. The increased ductility of LUHPC is particularly advantageous in these contexts, as it can absorb more strain energy without failing prematurely.

The OS-LUHPC-CBD with a 12 mm steel deck plate exhibited improved ductile performance over the 16 mm configuration, showing that decreasing the steel deck plate thickness enhanced the composite system’s energy dissipation capacity. The OS-LUHPC-CBD specimens with the 12 mm steel deck plate configuration also demonstrated marginally better deformation capacity than OS-LUHPC-CBD specimens with 16 mm plates.

Generally, the ductile performance of both the OS-LUHPC-CBD and OS-UHPC-CBD systems was characterized by significant post-peak deformation and energy dissipation. The LUHPC slab showed improved ductility due to its lower density and enhanced flexibility. The results generally indicate that the proposed lightweight OS-LUHPC-CBD with a 12 mm steel deck exhibits sufficient deformation capacity to be used in real-world bridge applications. It offers the benefits of better deformation tolerance and energy dissipation, which are crucial for bridge decks’ long-term durability and serviceability.

### 3.4. Load–Interfacial Slippage

**Figure 17:**
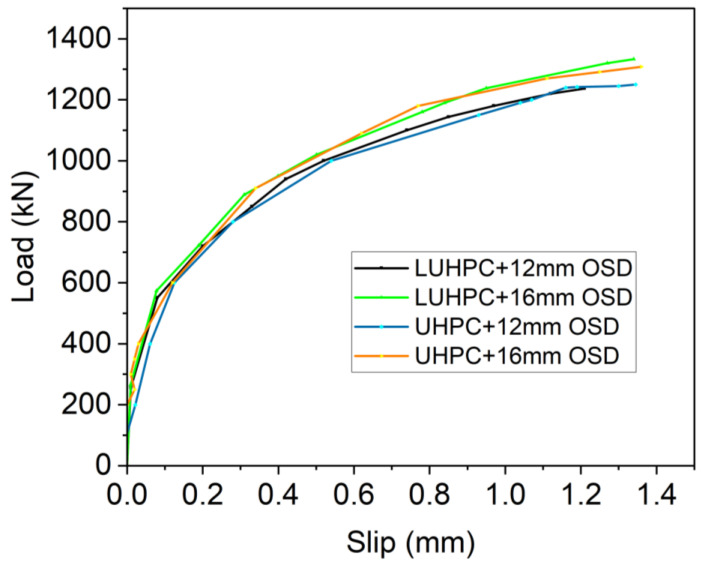
Interfacial slip between the steel deck and the respective slabs of OS-LUHPC-CBD and OS-UHPC-CBD systems.

The interfacial slippage behavior of both the OS-LUHPC-CBD and the OS-UHPC-CBD systems was evaluated by measuring the relative displacement at both ends of each specimen. The average slippage values were recorded and plotted against the applied load to construct the load–slippage curves, as shown in Figure 17. All specimens exhibited similar slippage trends, indicating the consistent shear transfer behavior of the specimens from the orthotropic steel deck to the concrete slabs and vice versa.

The load–slip curve trend, as can be observed from Figure 17, shows that as the load increases, the slip between the steel LUHPC and UHPC slabs gradually develops, demonstrating a nonlinear progression. The curves for all specimen configurations are closely aligned, suggesting consistent interfacial behavior regardless of the slab material type or steel deck thickness, with slip values remaining within a narrow range even under high loads approaching ultimate flexural capacity. These results are similar to findings from previous studies on UHPC–steel composite systems, where the shear studs’ performance limited excessive slippage and ensured adequate composite action [17]. The consistency in slippage in all specimens can be attributed to the design of the specimens based on a normalized stud number (α = 1.0) where the shear studs exhibit high slip resistance, ensuring effective load transfer and minimizing premature interfacial failure.

Despite the observed positive composite behavior of the OS-LUHPC-CBD and the OS-UHPC-CBD systems, the shear slippage observed highlights the importance of optimizing shear connector design to enhance long-term performance. Further research on alternative shear connection mechanisms, such as hybrid connectors or surface treatments, is required to reduce interface movement, especially under cyclic loading conditions common in bridge applications [53,54].

### 3.5. Strain Distribution

**Figure 18:**
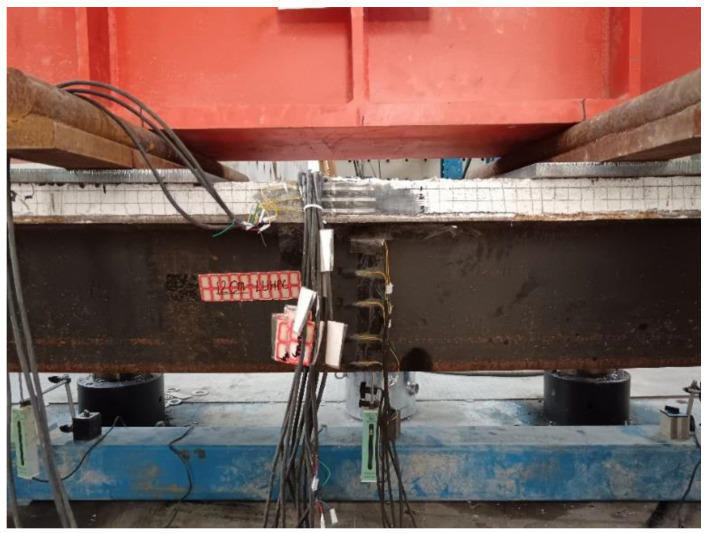
Strain gauges placed at the mid-span on the steel deck, trapezoidal ribs, and slabs.

**Figure 19:**
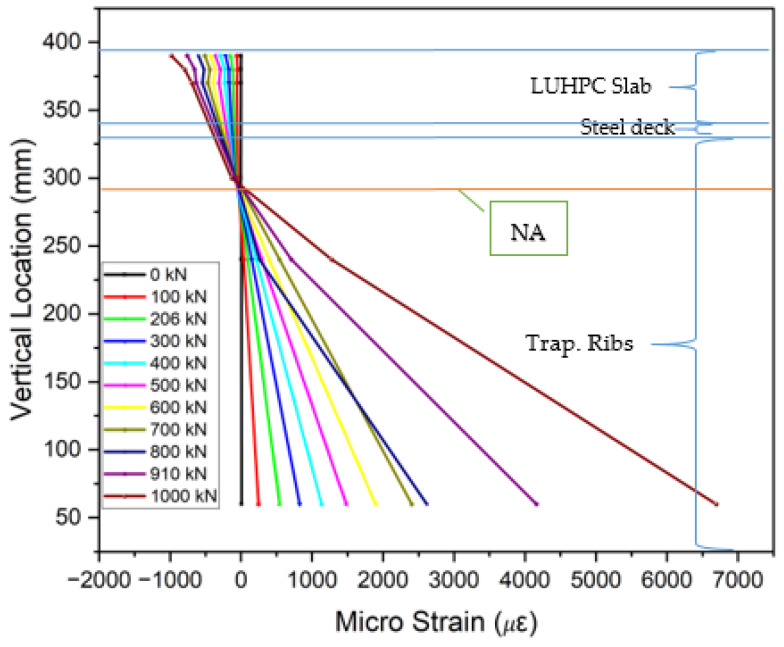
Strain along the mid-span section of OS-LUHPC-CBD (12 mm steel deck).

**Figure 20:**
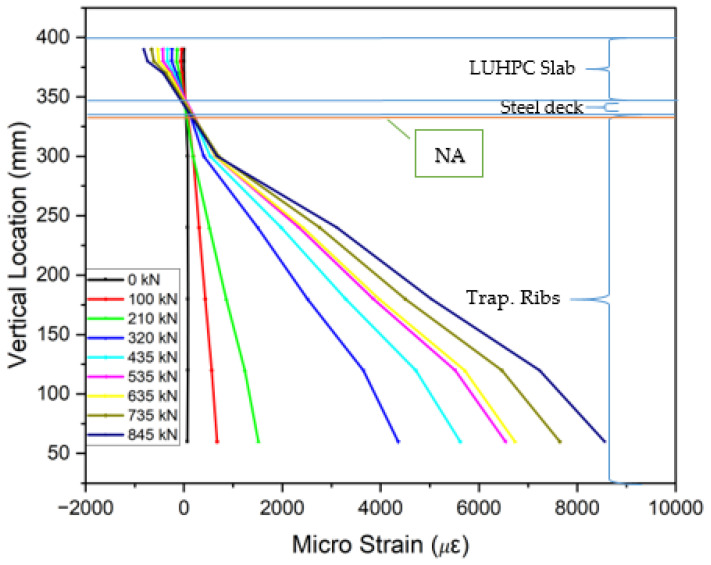
Strain along the mid-span section of OS-LUHPC-CBD (16 mm steel deck).

**Figure 21:**
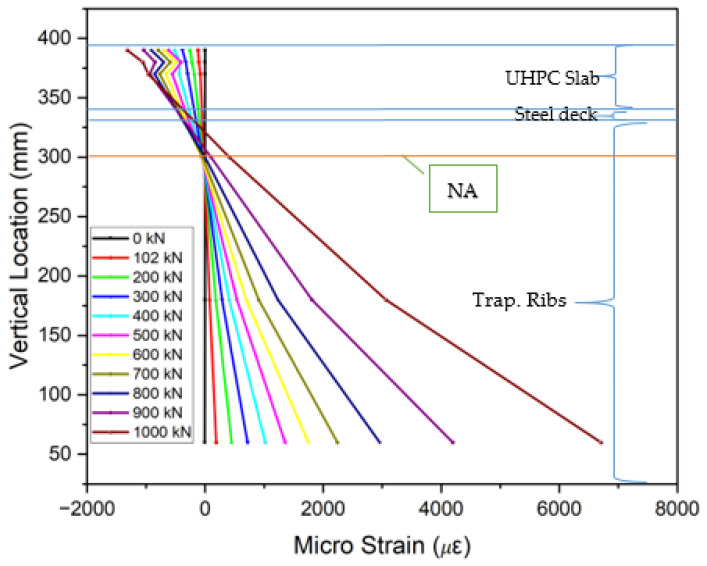
Strain along the mid-span section of OS-UHPC-CBD (12 mm steel deck).

**Figure 22:**
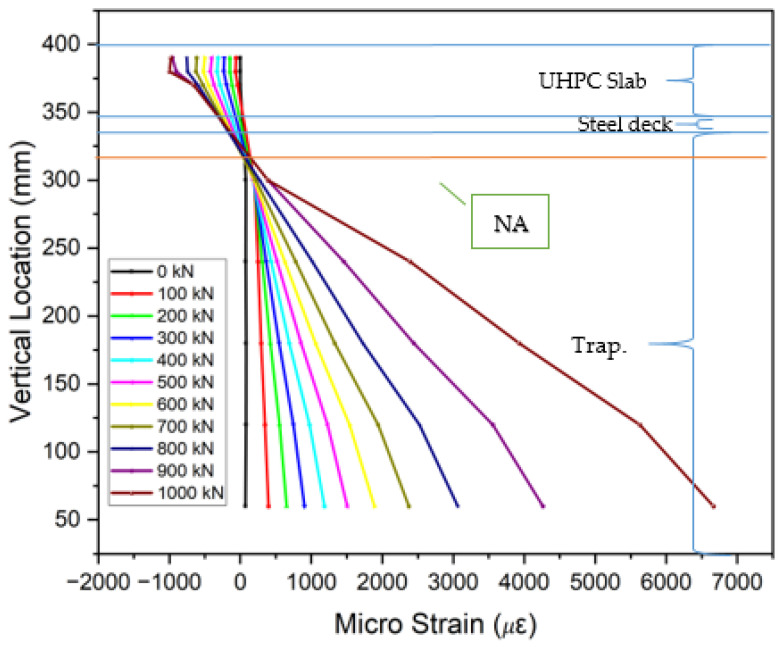
Strain along the mid-span section of OS-UHPC-CBD (16 mm steel deck).

The collected strain data from gauges placed on the steel decks, trapezoidal ribs, and UHPC or LUHPC slabs, as displayed in Figure 18, show that the neutral axis remained within the orthotropic steel deck. An analysis of the strain distribution across all OS-LUHPC-CBD and OS-LUHPC-CBD systems with 12 mm and 16 mm steel decks, as illustrated in Figure 19, Figure 20, Figure 21 and Figure 22, displays a clearly defined neutral axis (NA), signifying the transition between the compressive forces in the LUHPC and UHPC slabs and the steel deck and the tensile forces in the trapezoidal ribs. With increased load application, the compressive strain in the slabs stays relatively moderate, indicating their effective load distribution. The trapezoidal ribs exhibited a considerable increase in tensile strain, underscoring its very important role in the systems’ tension resistance and the importance of its optimization. This finding shows that, as the primary tension-carrying element, the trapezoidal rib must be optimized at rib-to-deck (RD) and rib-to-floor beam (RF) connections to prevent fatigue cracks and ensure efficient load transfer. Additionally, the slenderness ratio between the rib and the steel deck must also be carefully considered.

All of the specimens displayed comparable strain profile behaviors, characterized by a gradual and consistent increase in strain magnitudes along the loading processes. The absence of abrupt variations in strain measurements between the steel deck and the slabs suggests that the shear connection successfully avoided substantial interfacial slippage, which indicates that the bonding and load transfer capacity of LUHPC and UHPC slabs are sufficient.

## 4. Numerical Analysis

### 4.1. Establishment of Finite Element Model

To numerically investigate the longitudinal flexural behavior of the OS-LUHPC-CBD and OS-UHPC-CBD systems, three-dimensional finite element (FE) models were developed using ABAQUS CAE. These models were constructed based on the detailed drawings shown in Figure 2, the same as the specimens under the experimental investigation simulating the configurations of steel decks with 12 mm, 14 mm, and 16 mm thicknesses, closed trapezoidal ribs, and 50 mm thick slabs of reinforced LUHPC and UHPC materials connected to the steel deck via shear studs. A four-point bending test was undertaken on the developed finite element model to investigate both the flexural behavior and parametric influences on the systems’ flexural performance.

**Figure 23:**
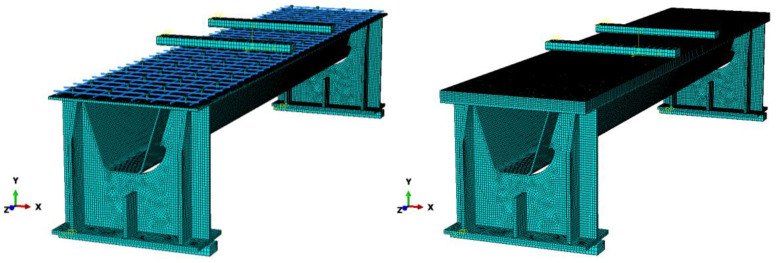
The finite element model of the OS-LUHPC-CBD and OS-UHPC-CBD.

The finite element model consists of six major components: the steel deck plate, shear studs, the crossbeam, trapezoidal ribs, steel reinforcement, and the LUHPC or UHPC slabs, as shown in Figure 23. For the accurate representation of the structural components, eight-node hexahedral solid elements (C3D8R) were used for the steel deck, trapezoidal ribs, crossbeams, shear studs, and UHPC and LUHPC slabs, while truss elements (T3D2) were applied to simulate the steel reinforcement. Mesh refinement was applied to regions with high-stress concentrations to capture local deformations, including buckling. The adaptive ALE (Arbitrary Lagrangian–Eulerian) method was used to simulate the cracking and crushing behavior of the LUHPC and UHPC slabs for more accurate damage progression predictions.

To capture the nonlinear behavior and damage evolution of both LUHPC and UHPC materials, the concrete damage plasticity (CDP) model was implemented. The stress–strain curves for both materials, adapted from Graybeal (modified) [55], were used as input for defining material behavior under tension and compression. The damage variables (*d_c_* for compression and *d_t_* for tension) were defined as functions of plastic strain and represented the degradation of stiffness as the materials approached failure. The model accounts for tensile and compressive damage and provides a reliable method for simulating material degradation during loading.

**Figure 24:**
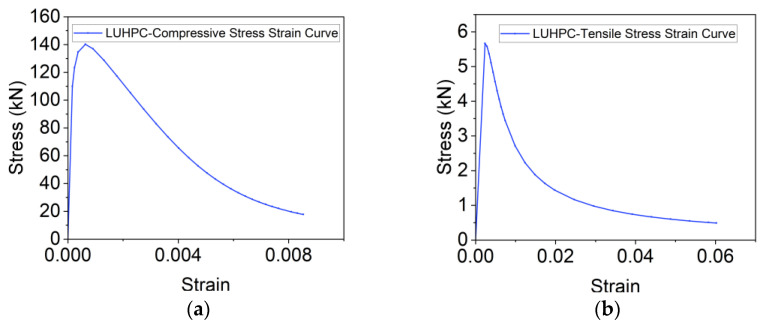

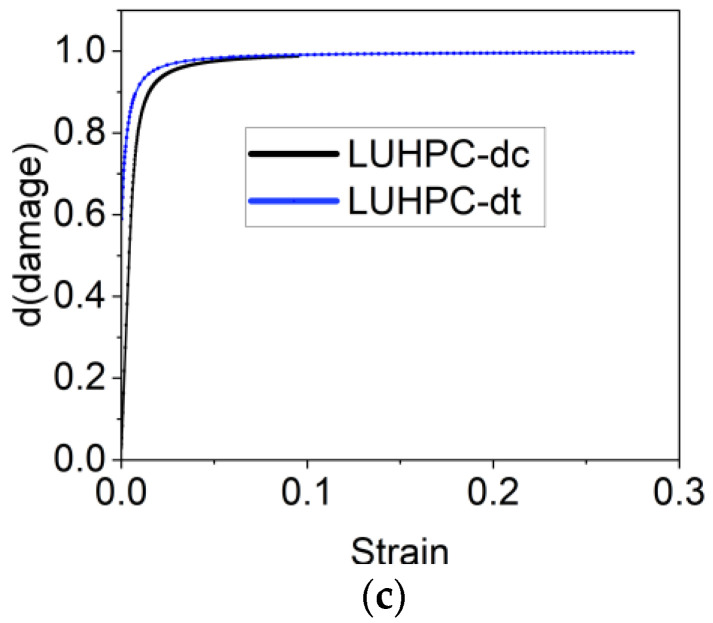
Constitutive relationships of LUHPC for (**a**) compression, (**b**) tension, and (**c**) damage.

**Figure 25:**
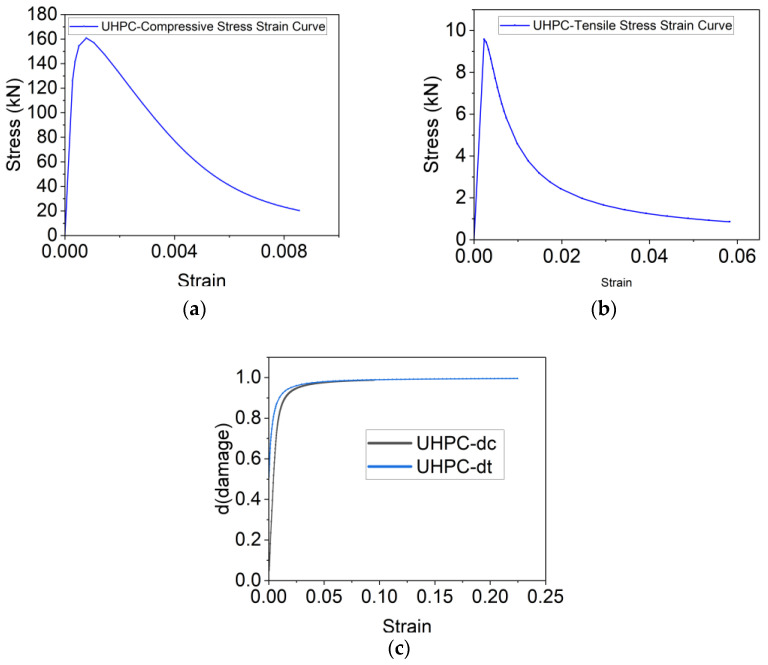
Constitutive relationships of UHPC for (**a**) compression, (**b**) tension, and (**c**) damage.

The constitutive relationships [56,57] for LUHPC and UHPC, along with their damage curves, are illustrated in Figure 24 and Figure 25. The parameters for density, elastic–plastic properties, and plastic flow are presented in Table 6 and Table 7. The damage parameters for tension and compression (*d_t_* and *d_c_*) were selected based on Pavlović et al. [58].

**Figure 26:**
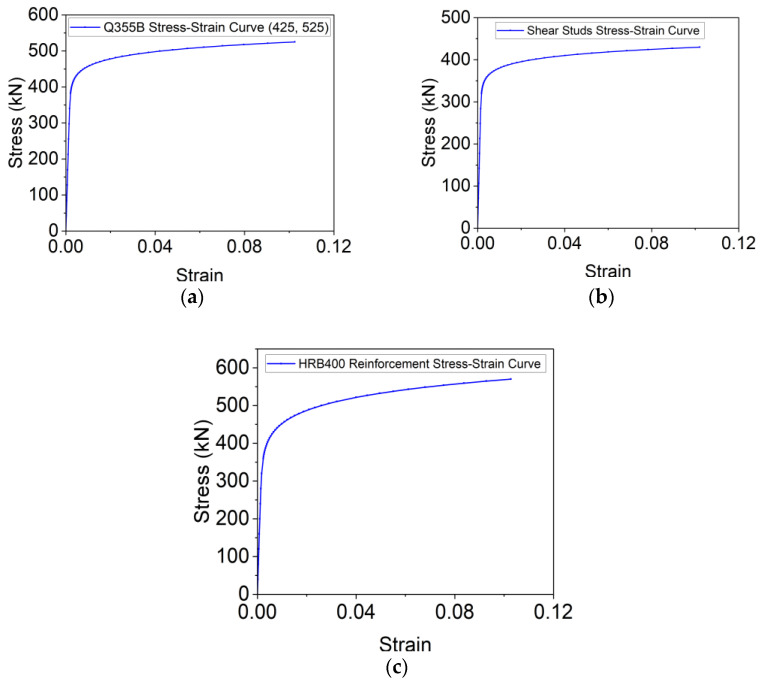
Constitutive relationships of (**a**) steel plates, (**b**) shear studs, and (**c**) rebars.

Since cracking is not expected in a steel deck plate, reinforcement bars, or shear studs during loading, an ideal elastic–plastic material model was used to simulate the steel components. The trilinear stress–strain model for steel plates, shear studs, and rebars includes the yield strength (*f_y_*) and ultimate strength *f_u_*). This model, which simplifies the mechanical behavior of steel components, provides satisfactory results and is commonly used in modeling steel structures under static loading conditions [34,35]. The constitutive relationships for the steel components are shown in Figure 26, and the corresponding material properties are summarized in Table 4.

The loading was applied as a vertical displacement at the rigid part of the model, coupled with reference points (RPs) placed on the top surfaces of the steel deck plate. This approach ensures stability and convergence throughout the analysis by avoiding direct force loading. Contact properties between the steel deck plate and LUHPC and UHPC slabs were modeled using hard contact and penalty algorithms, with a friction coefficient of 0.4 applied to the steel–concrete interfaces.

### 4.2. Model Validation

**Figure 27:**
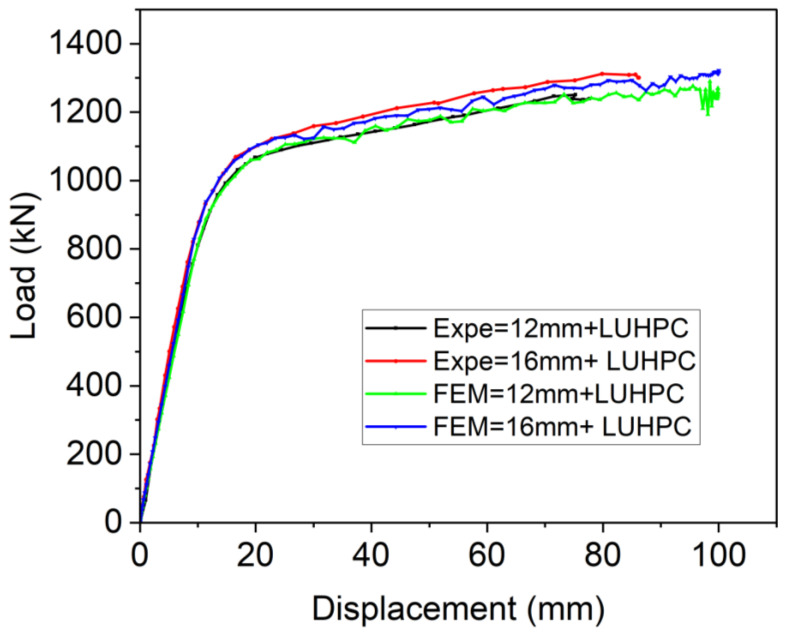
Comparison of experimental and numerical results of load–deflection response of OS-LUHPC-CBB (12 mm and 16 mm steel deck).

**Figure 28:**
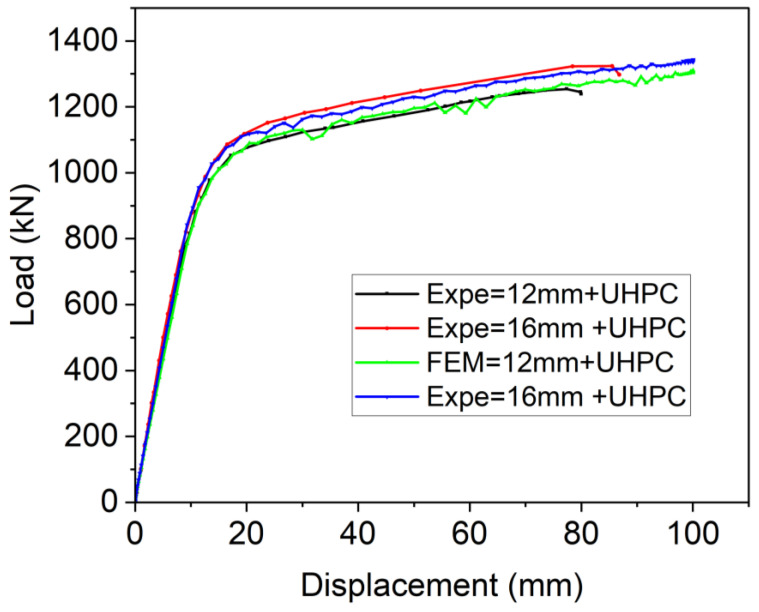
Comparison of experimental and numerical results of load–deflection response of OS-UHPC-CBB (12 mm and 16 mm steel deck).

**Figure 29:**
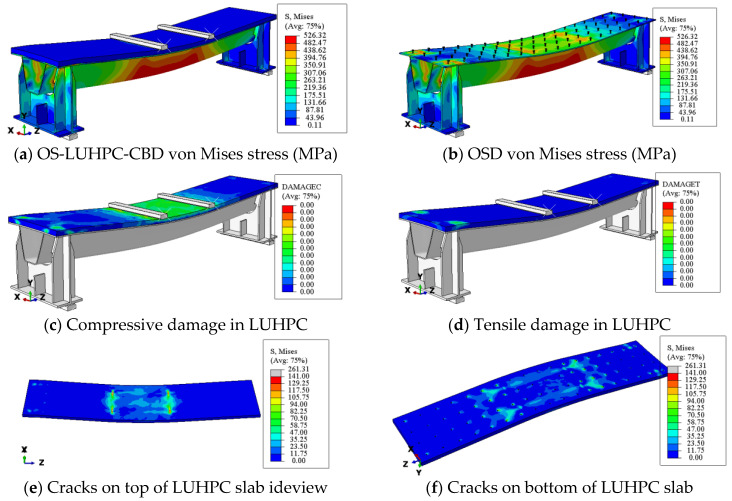
Finite element analysis results for the proposed lightweight OS-LUHPC-CBB with 12 mm steel deck.

To ensure the accuracy and reliability of the developed finite element model, its predictions of flexural strength and failure modes were compared against experimental data from four-point bending tests of OS-LUHPC-CBD and OS-UHPC-CBD specimens with closed trapezoidal ribs, 12 mm and 16 mm steel deck plates, 50 mm LUHPC slabs, and 50 mm UHPC slabs. The comparison of the numerical and experimental load–deflection curves of these specimens, shown in Figure 27 and Figure 28, revealed a strong correlation, particularly during the linear loading phase. The numerical analyses of the flexural performance of the proposed OS-LUHPC-CBB and OS-UHPC-CBD with 12 mm and 16 mm steel decks are presented in Figure 27 and Figure 28. Cracks and damage on the LUHPC slab and the stress distribution of the proposed OS-LUHPC-CBD system are presented in detail in Figure 29.

The model demonstrated minimal discrepancy in predicting ultimate load values, with errors of approximately 0.78% and 0.91% for the OS-LUHPC-CBD with both the 12 mm and 16 mm steel deck plates and 0.85% and 0.93% for the OS-UHPC-CBD with the same plate thicknesses, respectively. These results confirm that the finite element model is precise enough to predict the flexural behavior of the composite deck systems, making it reliable for further parametric analyses and design optimization.

### 4.3. Parametric Study

This study also investigated the influence of various design parameters on the flexural behavior of the proposed lightweight OS-LUHPC-CBD and the OS-UHPC-CBD. The parameters under study included key geometric factors such as the thickness of the steel deck plate, the rib, and the LUHPC or UHPC slabs. The effect of these parameters on flexural capacity was evaluated using a validated finite element model. Nine finite element models were developed based on the validated model, all with identical section sizes and reinforcement details.

The following geometric design parameters and their respective values listed in Table 8 were considered in the parametric study.

**Figure 30:**
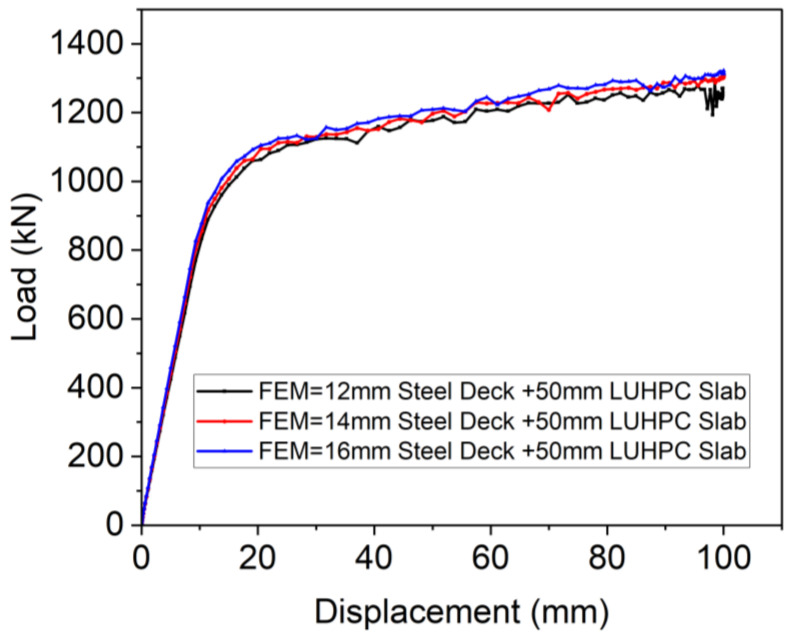
Effect of steel deck thickness on OS-LUHPC-CBD’s flexural performance.

**The effect of the steel deck thickness:** The results of the parametric study showed that the ultimate load capacity (*P_u_*) of the OS-LUHPC-CBD system with a 50 mm LUHPC slab increased with increasing steel deck plate thickness, as can be seen from Figure 30. For example, the ultimate load for the OS-LUHPC-CBD system was 1221.83 kN, 1280.3 kN, and 1323.25 kN for 12 mm, 14 mm, and 16 mm steel deck thicknesses, respectively. The system showed a decrease in ultimate load capacity of 4.56% and 3.25% as the steel plate thickness decreased from 14 mm to 12 mm and from 16 mm to 14 mm, respectively.

**Figure 31:**
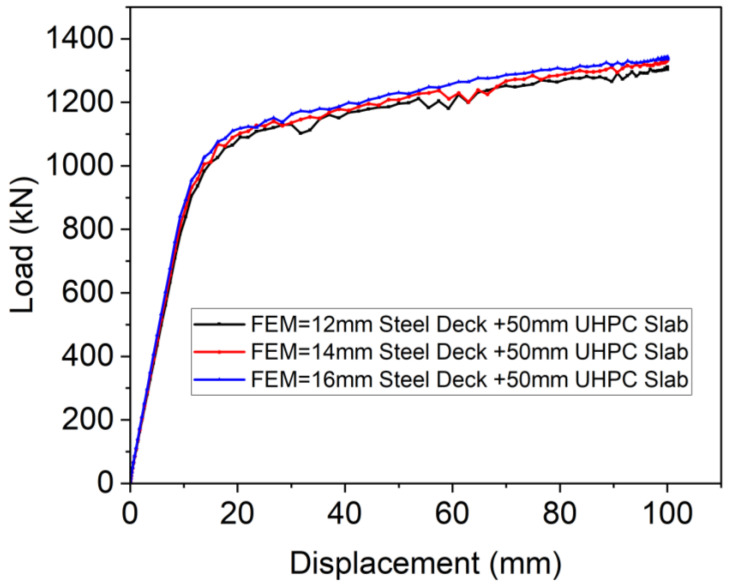
Effect of steel deck thickness on OS-UHPC-CBD’s flexural performance.

Similarly, as can be seen in Figure 31, the OS-UHPC-CBD system showed identical behavior to that depicted in Figure 29, with ultimate loads of 1231.7 kN, 1290.4 kN, and 1336.25 kN for 12 mm, 14 mm, and 16 mm steel deck thicknesses, respectively. The flexural capacity of the OS-UHPC-CBD system decreased by approximately 4.76% and 3.55% when the thickness of the deck plate was reduced from 14 mm to 12 mm and from 16 mm to 14 mm, respectively.

In conclusion, the thickness of the steel deck plate significantly influences the ultimate load capacity of both the OS-LUHPC-CBD and OS-UHPC-CBD systems. These findings highlight the importance of optimizing deck plate thickness in designing these systems to enhance structural performance and load-bearing capacity.

**The effect of slab type and thickness:** The effect of slab type and thickness on the ultimate load capacity of the composite bridge decks was analyzed using the finite element model. Both the LUHPC and UHPC slabs contributed to mitigating local buckling under compressive stress, thereby enhancing load distribution over the steel deck plate, especially for the 12 mm steel deck plates.

**Figure 32:**
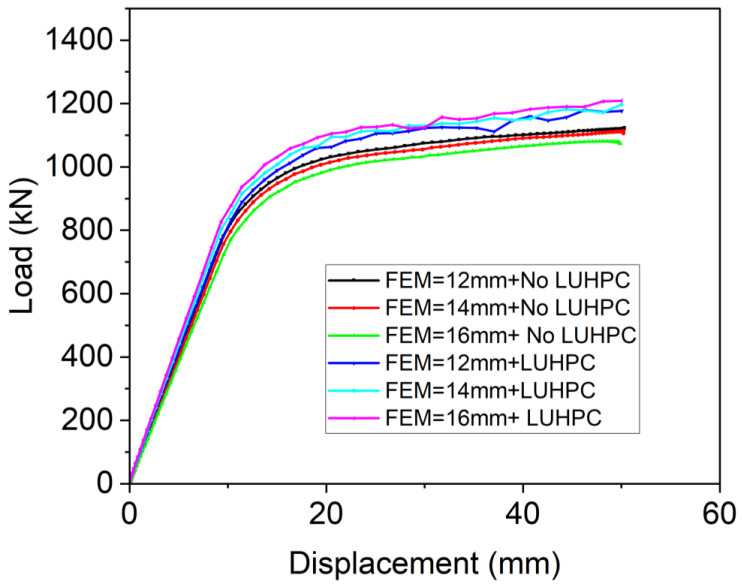
Contribution of the LUHPC slab to the OS-LUHPC-CBD on the ultimate load-bearing capacity of the OSD.

The orthotropic steel deck (OSD) system, which does not have either LUHPC or UHPC composite slabs, exhibited ultimate load capacities of 1037.7 kN, 1107.4 kN, and 1198.2 kN for the 12 mm, 14 mm, and 16 mm steel deck thicknesses, respectively. The LUHPC slabs improved the ultimate load capacity mentioned above by 8.74%, 8.61%, and 8.43%, respectively, while the UHPC slabs contributed 9.18%, 9.24%, and 9.31%, as can be clearly observed in Figure 32, for the 12 mm, 14 mm, and 16 mm slabs. These comparable enhancement levels suggest that the performance benefits of incorporating LUHPC or UHPC are maintained across a range of deck plate thicknesses and, importantly, that reducing the thickness of the steel deck plate to 12 mm is feasible without a significant compromise in load-bearing capacity, offering potential material savings.

Further analysis of the effects of slab thickness within the composite systems revealed that increasing the thickness of the LUHPC or UHPC slab yields measurable improvements in flexural capacity. For the OS-LUHPC-CBD system with a 12 mm steel deck plate, increasing the LUHPC slab thickness from 50 mm to 60 mm resulted in a 1.41% increase in the ultimate load capacity, with a further increase of 1.38% when the thickness was increased from 60 mm to 70 mm. A similar trend was observed for the OS-UHPC-CBD system, where corresponding increases of 1.44% and 1.57% were recorded. These incremental gains in load-bearing capacity indicate that thicker slabs significantly enhance the composite system’s overall stiffness and flexural performance.

The findings generally demonstrate that the thickness increment of both LUHPC and UHPC slabs have less effect in optimizing the structural efficiency of composite bridge decks when compared the steel deck and rib thickness. The results provide valuable insights for designing composite systems that aim to achieve high flexural performance while potentially reducing material usage.

**The effect of trapezoidal rib thickness:** The thickness of the ribs plays a critical role in determining the flexural capacity of composite bridge deck systems. The finite element analysis revealed that increasing rib thickness enhanced the ultimate load capacity by keeping the system’s slenderness ratio as low as possible to improve the load distribution across the composite structure and enhance the resistance to local buckling, thereby increasing the overall stiffness.

For the proposed OS-LUHPC-CBD system comprising a 12 mm steel deck plate and a 50 mm LUHPC slab, increasing the rib thickness from 8 mm to 9 mm resulted in a 2.30% improvement in flexural capacity. A further increase from 9 mm to 10 mm produced a 2.72% enhancement. Similarly, the OS-UHPC-CBD system, which utilizes a 12 mm steel deck plate with a 50 mm UHPC slab, exhibited flexural capacity gains of 2.66% and 2.53% for the corresponding rib thickness increments.

The observed improvements can be seen from the perspective of the effect of rib thickness keeping the slenderness ratio to a minimum. A thicker rib increases the slenderness ratio, negatively impacting the system and creating local effects. Notably, these effects are most pronounced when the steel deck plate is relatively thin (12 mm).

Optimizing rib thickness is essential for maximizing composite bridge decks’ flexural performance and stability. These findings provide an insight into enhancing structural efficiency while mitigating the slenderness ratio and local buckling effect, particularly in designs employing thinner steel deck plates.

The slenderness ratio, in the form of L/r, where L is the unsupported length and r is the radius of gyration, becomes a governing parameter in the design of longitudinal ribs. To prevent overall buckling of the deck induced by girder bending, the slenderness ratio of a longitudinal rib should not exceed the limit prescribed by the specification [38]. While increasing the rib thickness can enhance structural performance, it may also inadvertently result in a higher slenderness ratio if the rib geometry is not adjusted proportionally.

The findings from this parametric study offer insights into the design of the OS-LUHPC-CBD and OS-UHPC-CBD systems, highlighting the importance of the strategic selection of geometric parameters to achieve a balance between load-bearing capacity, stiffness, and material efficiency. The results can be used to guide the optimization of lightweight composite bridge deck design, integrating parameters for high-performance and cost-effective solutions in real-world applications.

## 5. Conclusions

In this research, an OS-LUHPC-CBD system was proposed that meets the requirements of high performance, light weight, and low cost. Experimental and numerical analyses were performed on the OS-LUHPC-CBD and OS-UHPC-CBD systems, yielding significant insights into the flexural performance and optimization of the systems. The following conclusions are drawn:

1. The experimental results demonstrated that the proposed OS-LUHPC-CBD has equivalent flexural capacity and improved ductility compared to the OS-UHPC-CBD, and is primarily characterized by flexural failure, as evidenced by the mid-span crushing of the LUHPC and UHPC slabs and yielding of the trapezoidal rib. The OS-LUHPC-CBD showed ductile flexural behavior under ultimate loads with the advantages of being 8.4% lighter in weight and 6.8% lower in cost while being just as easy to construct.
2. The findings from this experimental investigation also show that when the steel deck thickness decreased from 16 mm to 12 mm in both the OS-LUHPC-CBD and OS-UHPC-CBD specimens, it resulted in a 6.2% and 5.9% decrease in the ultimate load-bearing capacity and 8.6% and 7.9% reduction in yield load, respectively, with no physically observable differences in their response upon ultimate load application.
3. A finite element model with the use of concrete damage plasticity (CDP) was developed and validated, and showed strong correlation with the experimental data in predicting load–deflection, thus proving to be a reliable tool to numerically assess the performance of the OS-LUHPC-CBD and OS-UHPC-CBD systems.
4. The parametric study conducted as part of the numerical analysis revealed the significant influence that important geometric parameters have, such as steel deck thickness, rib web thickness, and LUHPC and UHPC slab thickness. Increases in these thicknesses may increase the yielding load and ultimate load and their corresponding deformations.
5. This research highlights the potential of the OS-LUHPC-CBD system as a transformative solution for use in long-span bridge decks, offering enhanced flexural performance with reduced material usage and addressing performance and economic concerns in modern infrastructure. Aside from the mentioned mechanical performance results, LUHPC has transformative potential for the design of long-span bridges through the facilitation of lower dead loads, remarkable durability, and novel structural designs. However, to guarantee practical viability, challenges related to large-scale implementation, such as the absence of standardization, need to be addressed. Future studies should focus on creating guidelines to facilitate industry adoption.

## Figures and Tables

**Table 1:** LUHPC mix design (kg/m^3^) [25].

Cement	Clay	Silica Fume	Fly Ash Microbeads	Water Reducing Agent	Water	Steel Fiber
810	667	200	190	34	204	160

**Table 2:** UHPC mix design (kg/m^3^).

Cement	Fly Ash Microbeads	Silica Fume	Quartz Sand (Mesh)			Steel Fiber	Water Reducing Agent	Expanding Agent	Water
			20–40	40–80	80–120				
760	200	160	400	350	200	200	22	100	180

**Table 3:** Material properties of LUHPC and UHPC.

Concrete Type	Density kg/m^3^	Tensile Strength MPa	Compressive Strength MPa	Young’s Modulus GPa	Poisson’s Ratio
LUHPC	2045	5.65	139	41.75	0.2
UHPC	2800	9.56	161	50.1	0.2

**Table 4:** Material properties of the steel, reinforcement, and headed steel stud.

Steel Type	Standard	Yield Strength MPa	Ultimate Strength MPa	Young’s Modulus Gpa	Poisson’s Ratio
OSD plates	Q425	425	525	206	0.3
Reinforcement	HRB400	400	570	206	0.3
Headed studs	h = 35 mm dia = 13 mm	425	525	206	0.3

**Table 5:** Deflection ductility coefficient of the specimens.

Specimen	Yield Deflection (mm)	Ultimate Deflection (mm)	Deflection Ductility Coefficient μΔ
OS-LUHPC-CBD (12 mm steel deck)	17.2	84.49	4.912
OS-LUHPC-CBD (16 mm steel deck)	16.3	78.43	4.813
OS-UHPC-CBD (12 mm steel deck)	16.01	77.4	4.834
OS-UHPC-CBD (16 mm steel deck)	16.91	68.18	4.032

**Table 6:** Density and elastic–plastic parameters of the LUHPC and UHPC.

Concrete	Density kg/m^3^	Tensile Strength Mpa	Compressive Strength Mpa	Young’s Modulus Gpa	Poisson’s Ratio
LUHPC	2045 (Ding et al., 2019) [24]	5.65	139	41.75	0.2
UHPC	2800	9.58	160.8	50.1	0.2

**Table 7:** Concrete damage plasticity (CDP) parameters of the LUHPC and UHPC.

Concrete	Dilation Angle	Eccentricity	fb_0_/fc_0_	k	Viscosity
LUHPC	51	0.1	1.1	0.66	0
UHPC	54	0.1	1.1	0.66	0

**Table 8:** Key geometric design parameters considered in the parametric study.

Design Parameter	Symbol	Values Considered (mm)
Steel deck plate thickness (mm)	*t_s_*	12, 14, 16
Trapezoidal rib thickness (mm)	*t_h_*	8–10
UHPC slab thickness (mm)	*t_s_*	50, 60, 70

## Data Availability

The raw data supporting the conclusions of this article will be made available by the authors on request.

## Acronyms

ALE	Arbitrary Lagrangian–Eulerian
CDP	Concrete Damage Plasticity
C3D8R	8-Node Hexahedral Solid Elements
LUHPC	Lightweight Ultra-High-Performance Concrete
NA	Neutral Axis
OSDs	Orthotropic Steel Decks
OSBDs	Orthotropic Steel Bridge Decks
OS-LUHPC-CBD	Orthotropic Steel Lightweight Ultra-High-Performance Composite Bridge Deck
OS-UHPC-CBD	Orthotropic Steel Ultra-High-Performance Composite Bridge Deck
RD	Rib to Deck Connection
RF	Rib to Floor Beam Connection
RPs	Reference Points
UHPC	Ultra-High-Performance Concrete
